# CD73 Overexpression in Podocytes: A Novel Marker of Podocyte Injury in Human Kidney Disease

**DOI:** 10.3390/ijms22147642

**Published:** 2021-07-16

**Authors:** Zoran V. Popovic, Felix Bestvater, Damir Krunic, Bernhard K. Krämer, Raoul Bergner, Christian Löffler, Berthold Hocher, Alexander Marx, Stefan Porubsky

**Affiliations:** 1 Institute of Pathology, University Medical Centre Mannheim, University of Heidelberg, 68167 Mannheim, Germany; alexander.marx@umm.de; 2 Light Microscopy Facility, German Cancer Research Center, 69120 Heidelberg, Germany; f.bestvater@Dkfz-Heidelberg.de (F.B.); D.Krunic@dkfz-heidelberg.de (D.K.); 3 5th Department of Medicine (Nephrology, Hypertensiology, Endocrinology, Rheumatology), University Hospital Mannheim, University of Heidelberg, 68167 Mannheim, Germany; Bernhard.Kraemer@umm.de (B.K.K.); c.loeffler2@medius-kliniken.de (C.L.); Berthold.Hocher@medma.uni-heidelberg.de (B.H.); 4 Department of Rheumatology, Nephrology, Oncology, Klinikum Ludwigshafen, 67063 Ludwigshafen, Germany; BERGNERR@klilu.de; 5 Department of Internal Medicine, Rheumatology and Immunology, Medius Klinik, 73230 Kirchheim, Germany; 6 Institute of Pathology, University Medical Center, Johannes-Gutenberg University, 55131 Mainz, Germany

**Keywords:** podocyte, minimal change disease, CD73, CCR2

## Abstract

The CD73 pathway is an important anti-inflammatory mechanism in various disease settings. Observations in mouse models suggested that CD73 might have a protective role in kidney damage; however, no direct evidence of its role in human kidney disease has been described to date. Here, we hypothesized that podocyte injury in human kidney diseases alters CD73 expression that may facilitate the diagnosis of podocytopathies. We assessed the expression of CD73 and one of its functionally important targets, the C-C chemokine receptor type 2 (CCR2), in podocytes from kidney biopsies of 39 patients with podocytopathy (including focal segmental glomerulosclerosis (FSGS), minimal change disease (MCD), membranous glomerulonephritis (MGN) and amyloidosis) and a control group. Podocyte CD73 expression in each of the disease groups was significantly increased in comparison to controls (*p* < 0.001–*p* < 0.0001). Moreover, there was a marked negative correlation between CD73 and CCR2 expression, as confirmed by immunohistochemistry and immunofluorescence (Pearson r = −0.5068, *p* = 0.0031; Pearson r = −0.4705, *p* = 0.0313, respectively), thus suggesting a protective role of CD73 in kidney injury. Finally, we identify CD73 as a novel potential diagnostic marker of human podocytopathies, particularly of MCD that has been notorious for the lack of pathological features recognizable by light microscopy and immunohistochemistry.

## 1. Introduction

The evolutionary crucial role of adenosine triphosphate (ATP) as the ubiquitous intracellular energy source has been updated more than three decades ago with a revolutionary notion that the members of the purinergic system (ATP, adenosine di- and triphosphate (ADP and AMP, respectively) may as well deliver extracellular signals to mediate cellular interactions [1,2]. This concept led to the understanding that the purinergic system participates in shaping immune responses via modulation of receptor expression profile, cytokine secretion, production of reactive oxygen species (ROS) and intracellular pathogen digestion [3,4,5,6,7].

Extracellular ATP undergoes conversion/dephosphorylation into ADP and AMP via CD39 (ecto-nucleosidtriphosphate diphosphohydrolase 1) and finally, AMP is degraded to adenosine by CD73 (ecto-5′-nucleotidase) [8]. CD73 may be present in membrane-bound or soluble form. The membrane form has a dimer-structure consisting of two identical 70 kD subunits that bind to the plasma membrane via a link between C-terminal serine and glycosylphosphatidyl inositol (GPI) [9]. Noteworthy, CD39 substrates ATP and ADP appear to be competitive inhibitors of CD73 [9,10,11]. CD73 expression represents thus the critical pathway of tuning adenosine level in extracellular space. CD73 is not a cell or organ specific molecule; its expression on leukocytes, endothelium, epithelial or mesenchymal cells of various organs (kidney, lung, liver, colon, brain and heart) and stem cells has been well documented [12,13,14]. A protective role of adenosine via the nitric oxide (NO)-synthesis pathway in a liver ischemia-induced injury was first reported by Peralta et al. [15]. In concordance with these data, its action has been shown to ameliorate acute lung damage, allograft airway rejection, acute graft-versus-host disease and sepsis [4,16,17,18,19].

As convincingly demonstrated in several mouse models, CD73 may participate in tubulo-glomerular interaction in the kidney. CD73-mediated generation of adenosine derived by CD73 from ATP of tubular origin during ischemia-reperfusion damage has been shown to attenuate the inflammation via inhibition of the effector function of neutrophils [20]. Moreover, CD73-deficient mice show autoimmune inflammation that is accompanied by reduced numbers of podocytes and endothelial fenestrations [21]. This phenomenon was associated with increased serum levels of pro-inflammatory cytokines like tumor necrosis factor (TNF)-alpha or the chemokine C-X-C motif ligand 2 (CXCL-2).

In the setting of human kidney disease, it has been postulated that podocyte damage likely represents a mixture of adaptive and destructive mechanisms of cellular response to pathologic stimuli, not necessarily reflecting published observations from in vitro or animal models. The etiology of podocyte injury varies from mechanical stress (glomerular hyperfiltration), oxidative stress (like in hypertensive or diabetic nephropathy, toxic injury, inflammatory cell infiltration or animal models of minimal change disease) to immunologic stress (i.e., immune complex and complement mediated damage during glomerulonephritis) [22]. The inflammatory setting of glomerulonephritis can stimulate podocytes to synthesize various cytokines, potentially via CC chemokine receptor 2 (CCR2)-mediated signaling. Still, little is known on the role of CD73 in human kidney disease, especially in the setting of chronic inflammation and podocyte injury.

In the present study, we hypothesized that CD73 plays a role in the pathogenesis of human glomerular diseases and analyzed podocyte CD73 expression in human kidney biopsies from patients with podocytopathy-related pathologies. We demonstrate that podocyte surface expression of CD73 is a reliable marker of podocyte damage, thus in particular supporting the diagnostic value of CD73 in minimal change disease (MCD). Moreover, we report a negative correlation between podocyte CCR2 and CD73 expression, suggesting that overexpression of CD73 represents an adaptive response to glomerular lesion.

## 2. Results

### 2.1. Clinical Data and Ultrastructural Imaging of Podocyte Damage

**Figure 1:**
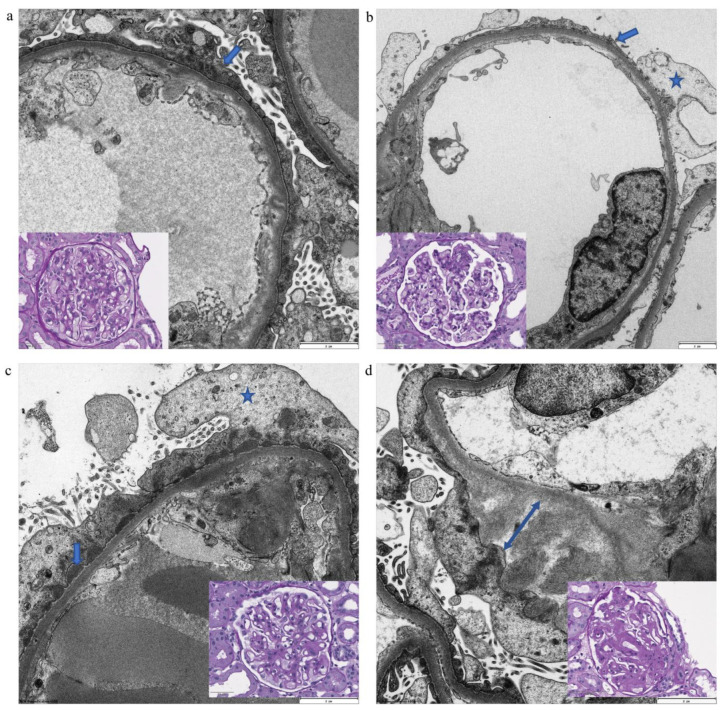
Ultrastructural image of primary and secondary podocyte damage. (**a**) Spreading of podocyte body on glomerular basement membrane (arrow) with retraction of foot processes in a case of MCD. (**b**) Extensive podocyte foot process effacement (arrow) and cytoplasmic vacuolization (star) in a patient with primary FSGS. (**c**) Foot process effacement (arrow), vacuolization and prominent organelles (double arrow) with underlying epimembranous electron-dense material (star) in primary membranous glomerulonephritis. (**d**) Subepithelial accumulation of amyloid-fibrils (two-head arrow) with consecutive podocyte detachment (as a sign of irreversible podocyte damage) in a patient with AL-amyloidosis (lambda). All electron micrographs are presented at 4000× magnification. Inserts—corresponding light microscopy photomicrographs (40×/0.65 NA; scale bar size: 50 µm in (**a**,**c**,**d**) and 100 µm in (**b**); PAS-staining).

Table 1 outlines the clinical presentation of cases included in the study regarding age, gender, creatinine and total proteinuria values. All FSGS patients had a primary NOS (not otherwise specified) form. Among 12 MGN patients, 5 were phospholipase A2 receptor (PLA2R)-positive (42%). In the amyloidosis group, 2 patients had AA-amyloidosis (16.7%) and 10 AL-amyloidosis (83.3%); in 2 patients with AL-amyloidosis light chain kappa was evident (20%) and 8 were positive for lambda (80% of AL-amyloidosis group). In all patients with MCD, FSGS, MGN and amyloidosis, podocyte damage was clearly detected by electron microscopy (Figure 1).

### 2.2. Surface Expression of CD73 Is Associated with Podocyte Injury in Human Kidney Disease

**Figure 2:**
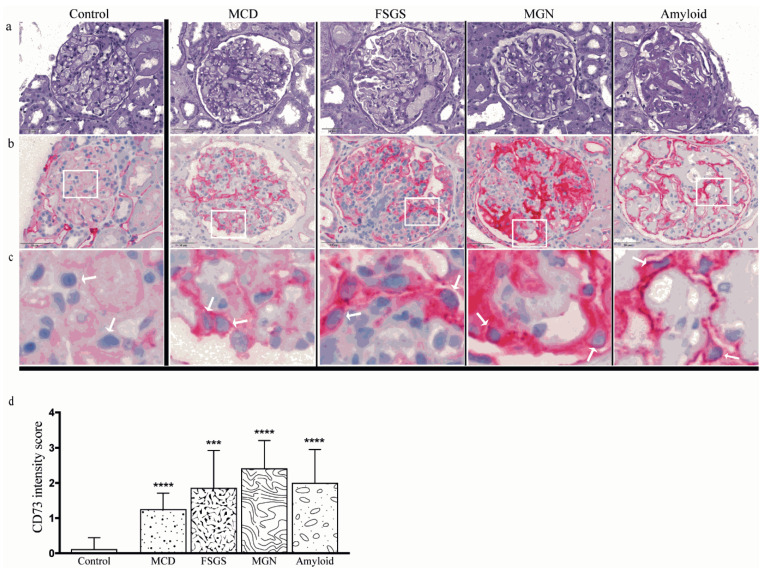
Surface expression of CD73 by injured podocytes. Negative CD73 immunohistochemistry in the control group (left column) in contrast to strong CD73 expression in primary and secondary podocytopathy groups (MCD—minimal change disease, FSGS—focal segmental glomerulosclerosis, MGN—membranous glomerulonephritis; (**a**) light microscopy photomicrographs, 40×/0.65 NA, PAS, (**b**) corresponding CD73 immunohistochemistry, 40×/0.65 NA, (**c**) 3× digital magnification from (**b**) with focus on podocytes (arrows). (**d**) Bar graph showing significant upregulation of podocyte expression in MCD, FSGS, MGN and amyloid groups in comparison to controls. Statistical differences were evaluated by unpaired *t*-test; ***, *p* < 0.001 and ****, *p* < 0.0001.

**Figure 3:**
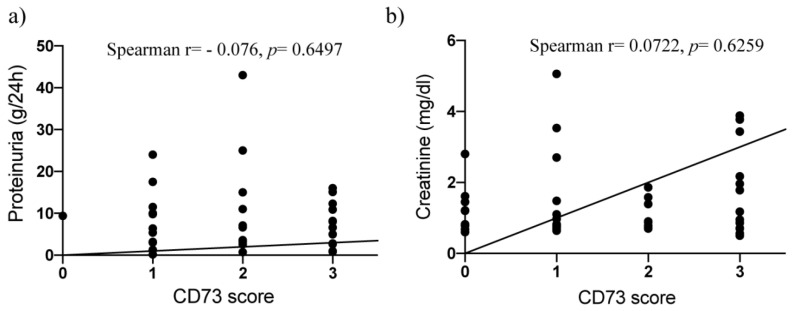
XY graph of correlation between CD73 score, (**a**) proteinuria (g/24 h) and (**b**) creatinine (mg/dL) levels. Spearman non-parametric correlation test; *n* = 38 (proteinuria—podocyte lesion group) and *n* = 48 (creatinine—all patients); two-tailed *p* value, confidence interval 95%. *p* > 0.05—no significant correlation.

To explore whether podocytes express CD73 in the physiological setting and to evaluate the pattern of CD73 in different disease settings related to podocyte damage, we performed immunohistochemical analysis of CD73 expression. Ultrastructural analysis confirmed podocyte damage as cytoskeletal irregularity with at least foot process effacement (Figure 1). In our negative control group without podocyte lesion, no significant podocyte expression of CD73 could be detected despite positivity of an internal control (peritubular capillaries) that typically stains positive (Figure 2). On the other hand, podocyte damage groups (MCD, FSGS, MGN and amyloidosis) showed a statistically highly significant upregulation of podocyte CD73 (*p* < 0.0001); the significant increase was consistent also when comparing single entities with negative controls (*p* at least < 0.001). Interestingly, the extent of CD73 upregulation tended to be lower in the MCD group, albeit significantly lower only in comparison with MGN samples (*p* = 0.0043) (Figure 2). In podocyte damage groups, there was no correlation between semiquantitative CD73 score and proteinuria on binary (nephrotic/non-nephrotic) or quantitative (g/24 h) level (Spearman r = −0.0807, *p* = 0.6302 and Spearman r = −0.076, *p* = 0.6497, respectively; Figure 3a). Overall, there was no correlation between CD73 score and creatinine level (Spearman r = 0.0722, *p* = 0.6259; Figure 3b). Additionally, we could not detect significant correlation between CD73 expression and hypertension (Spearman r = −0.03024, *p* = 0.8384) or diabetes mellitus (Spearman r = −0.1784, *p* = 0.2250).

### 2.3. Upregulation of Podocyte-Derived CD73 in Human Glomerular Disease Negatively Correlates with Expression of the CCR2 Receptor and Does Not Correlate with Nephrin and Podocin Expression

Appreciating published data on downregulation of macrophage CCR2 expression following CD73 overexpression in a stem cell model, we hypothesized that upregulation of CD73 during podocyte injury inversely correlates with expression of pro-inflammatory CCR2 and performed immunohistochemical CCR2 analysis. In the control group, no or only mild expression of CCR2 could be detected, without a significant discrepancy in comparison to the CD73 signature in the same samples. In contrast, mostly significant upregulation of surface CCR2 (yet of various intensity) could be seen in the majority of samples from podocyte-damage biopsies and was generally upregulated in comparison to the control group (*p* = 0.0206; Supplementary Figure S1). The disease groups did not significantly differ from each other in this regard.

**Figure 4:**
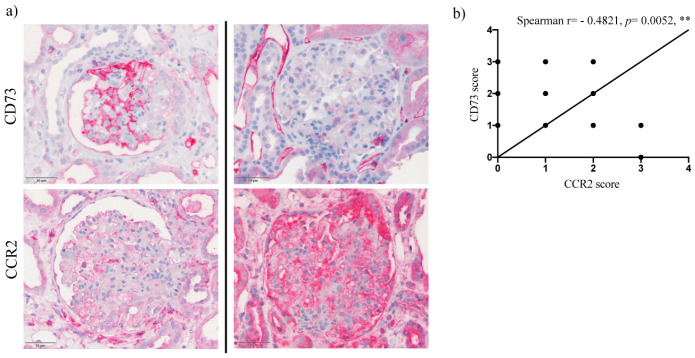
Correlation between podocyte CD73 and CCR2 expression by immunohistochemistry. (**a**) Photomicrographs showing immunohistochemistry for CD73 (upper row) and CCR2 (lower row) in two cases of MCD (left and right column). Stronger expression of CD73 is associated with milder positivity for CCR2 in podocytes, but also in surrounding cellular milieu (for example, parietal epithelial cells), 40×/0.65 NA. Scale bar = 50 µm. (**b**) Scatter plot with bars showing negative correlation between CD73 (squares) and CCR2 (triangles) intensity scores in podocyte damage groups (data are presented as mean ± SEM). The correlation was assessed by Spearman non-parametric correlation test (Spearman r = −0.4821; two-tailed *p* = 0.0052; ** significant negative correlation).

**Figure 5:**
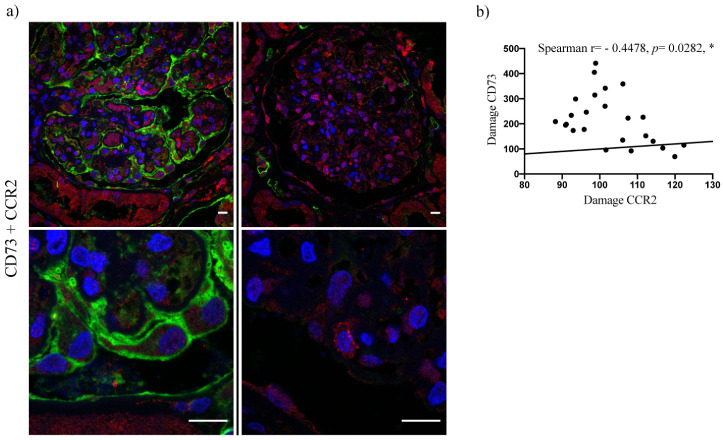
Inverse CD73/CCR2 correlation in podocytes by immunofluorescence. (**a**) CD73 (green signal) and CCR2 (red signal) co-staining in biopsy samples of two patients (left and right column). Images were acquired using the 60×/1.35 UPlanSApo objective. Bars = 10 µm. (**b**) Scatter plot with bars showing negative correlation between CD73 and CCR2 mean fluorescence intensity in podocytes (data are presented as mean ± SEM). The correlation was assessed by Spearman’s non-parametric test (Spearman r = −0.4478; *p* = 0.0282, * significant negative correlation).

**Figure 6:**
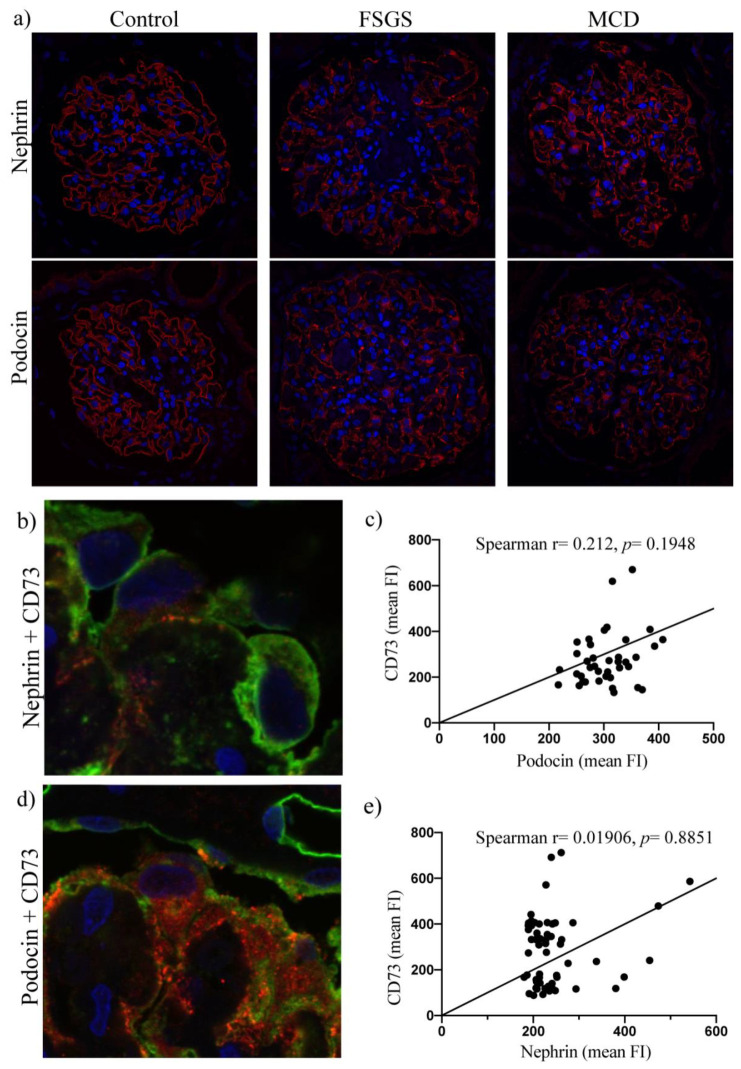
(**a**) In glomeruli with healthy podocytes, a uniform linear staining pattern of nephrin and podocin in the area of glomerular basement membrane can be seen (left). In contrast, glomeruli with damaged podocytes show a pathologic, granular staining pattern of podocyte-specific proteins (mid and right). (**d**) Co-stainings of podocyte-lesion samples show no significant correlation regarding mean fluorescence intensity ((**c**,**e**) Spearman non-parametric correlation test; *n* = 38; two-tailed *p* value, confidence interval 95%. *p* > 0.05—no significant correlation). CD73 (green)—nephrin (red) (**b**) and CD73 (green)—podocin (red).

We further analyzed a potential correlation between expression intensities of CD73 and CCR2. Indeed, comparing CD73 and CCR2 scores from the podocyte-injury biopsy cohort, a marked negative correlation could be observed (Spearman r = −0.4821, *p =* 0.0052), (Figure 4). Of note, glomeruli with CD73-positive podocytes tended to show reduced CCR2 expression not only in podocytes but also in surrounding cells, like parietal epithelial cells (Figure 4). The inverse correlation was confirmed by evaluation of immunofluorescent co-staining for CD73/CCR2 in damaged podocytes by confocal microscopy (Spearman r = −0.4478, *p =* 0.0282), (Figure 5). To address eventual association between CD73 positivity and (loss of) podocyte-specific protein expression, we performed additional immunofluorescent stainings for nephrin and podocin and evaluated their expression pattern in control, MCD and FSGS groups (Figure 6a) as well as co-staining with CD73 in samples with podocyte lesion (Figure 6b–e). We could not detect significant correlation between mean fluorescence intensity of podocin or nephrin expression on one side, and mean expression of CD73 on the other (*n* = 39, Spearman r = 0.212, *p* = 0.1948 for podocin and *n* = 60, Spearman r = 0.01906, *p =* 0.8851 for nephrin).

## 3. Discussion

We describe here for the first time the expression of CD73 on the surface of injured podocytes in human renal disease. Analyzing renal biopsies from patients with prominent podocyte damage such as in MCD, FSGS, MGN and amyloidosis, we report significant upregulation of podocyte CD73 expression in each of these groups in comparison to control kidneys, independent of the etiology of the podocyte damage. Moreover, we report an inverse correlation between CD73 and CCR2 expression by podocytes of podocyte-damage groups. Boosted podocyte-specific CCR2 has been shown to mediate diabetic renal injury in mice [23]. In addition, blockade of CCR2 in an Adriamycin-induced murine model of FSGS has been reported to reduce proteinuria and glomerular injury [24]. A recent study from Lee et al.—also using a mouse model—indicates that CCR2 depletion might ameliorate obesity-induced albuminuria via blockade of oxidative stress and lipid accumulation [25]. Indeed, in an in vitro system employing mesenchymal stem cells (MSC) from murine pericardial tissue, it has been shown that CD73-overexpressing MSCs dictate reparative processes via anti-inflammatory activity, among other factors also due to inhibition of CCR2-positive macrophage infiltration [13]. Hence, in the light of our results and published data, it is tempting to postulate that CD73 expression by podocytes may act protective in kidney injury, possibly via pathways that attenuate CCR2. CCR2 signaling is closely related to macrophage adhesion and activation [26,27]. As macrophages may influence the pathogenesis of podocytopathy-associated kidney diseases (like primary MGN, where infiltration of CD68^+^, CCR2^+^ macrophages has been recognized as a significant indicator of end-stage renal failure [28]), further studies of association between monocyte/macrophage content, origin (infiltrating vs. resident), activation profiles (M1—proinflammatory vs. M2—alternatively activated) and CD73 expression in human kidney disease are necessary to check the accordance of our results with previous data from murine models.

In an inflammatory micromilieu that can be seen in hypoxia, ischemia-reperfusion or autoimmune tissue damage multiple cells release members of the purinergic signaling system, predominantly in the form of pro-inflammatory ATP or ADP [29,30]. In these settings, ecto-nucleotidase CD73 and its product adenosine are important factors in immune response regulation and silencing. In animal models, CD73 has been shown to be protective in kidney ischemia reperfusion injury and its deficiency induced podocyte dysfunction, leading to autoimmune inflammation and kidney disease [21,31]. Other mouse in vivo experiments using ischemia reperfusion-injury revealed the important anti-inflammatory influence of CD73 expressed by proximal tubular epithelial cells and its crucial action in kidney protection during diabetic nephropathy via adenosine–endothelial Adora2b receptor signaling [20,32].

Podocytopathies are the most frequent group of glomerular lesions leading to proteinuria. MCD, a prototype ‘primary’ podocytopathy, is the most common cause of nephrotic proteinuria in children > 1 year of age [33,34]. Current hypotheses on pathogenesis of MCD suggest a significant role of cytokines and other immune mediators in disruption of podocyte-glomerular basement membrane integrity [35,36,37,38]. Discrepancy between absence of visible changes in light microscopy and severe proteinuria is the hallmark of MCD; ultrastructural evidence of podocyte damage by electron microscopy is thus a *conditio sine qua non* for the diagnosis of MCD to date. This diagnostic algorithm emphasizes the significance of further first-line diagnostic tools in this regard. The examination of CD73 expression, especially in the setting of limited availability of electron microscopy, might thus help to differentiate between glomerular (i.e., podocytopathic) and extraglomerular (e.g., tubular or paraproteinemic) sources of proteinuria. The comparison between immunohistochemistry and immunofluorescence in our study would speak in favor of the latter.

Several publications have convincingly documented aberrant distribution and loss of podocyte-specific proteins in various kidney disease settings that include podocyte injury and proteinuria, as recently reviewed [39]. In our study, within the podocyte-damage group, we could not detect a significant correlation between intensity of podocin/nephrin fluorescence and intensity of CD73 expression. However, specific limitations preclude pathophysiological interpretation of podocin/nephrin vs. CD73 statistical data. First, for the evaluation of fluorescence intensity we selected podocytes using technical magnification and resolution capacities of confocal microscopy. Although these conditions easily enable detection of podocyte bodies, major processes and especially foot processes remain indistinct, hence possibly not marked/analyzed. Second, correlation between expression of CD73 and podocyte-specific proteins in whole glomeruli cannot be justified: although assessment of podocin and nephrin at this level would be legitimate (as it is reasonable to assume that no other glomerular cells express these proteins) this cannot be claimed for CD73 which is partially expressed at least by endothelial cells. Third, evaluation of mean fluorescence intensity alone as independent marker of podocyte injury is questionable, as it does not cover the pathological, granular distribution pattern of podocin and nephrin within the glomerulus, which we could clearly observe in Figure 6. Therefore, we are reluctant to attribute the absence of CD73-podocin and CD73-nephrin correlation in our analysis to divergent molecular pathways of these markers. Further podocyte in vitro and animal studies are necessary to explore eventual interference between these signaling cascades.

Taken together, recognizing small sample size as the main limitation of our study, we demonstrate here a consistent CD73 overexpression by injured podocytes and suggest CD73 as a novel diagnostic marker of podocytopathy. In minimal change disease, our results may help to identify damaged podocytes and result in a less time- and cost-consuming diagnostic process. In addition, our results go in line with published data from murine experimental approaches and suggest a protective interaction between CD73 and CCR2 in human kidney disease. Further multicenter studies with larger patient cohorts are necessary to address mechanisms of CD73 signaling in human kidney disease and its prognostic value. Similarly, larger cohort numbers would be needed to establish a potential correlation between disease severity and CD73 expression.

## 4. Material and Methods

### 4.1. Study Cohort

A total of 49 biopsies included in this study from 49 different patients were diagnosed between 1 January 2018 and 31 December 2019 at the University Hospital Mannheim, University of Heidelberg. Further selection criteria were a representative biopsy with the possibility to perform triple-diagnostics with immunohistochemistry and EM, adequate clinical information, clear-cut diagnosis and sufficient material for this study. All patients were adult and Caucasians. The investigations enclosed 39 biopsies from patients with podocyte injury of varying etiologies (7 biopsies from patients with primary focal segmental glomerulosclerosis—FSGS; 8 from minimal change disease—MCD; 12 from patients with membranous glomerulonephritis—MGN; 12 from patients with amyloidosis). Negative controls were kidney biopsies from 10 patients without podocyte lesion and without proteinuria (6 patients with mild nephrosclerosis, 2 patients with interstitial nephritis and 2 patients without pathological changes). All of the above-mentioned biopsies were included in the analysis of podocyte CD73 expression; for CCR2, after exclusion of paraffin sections with insufficient number of non-sclerotic glomeruli (<5), we performed analysis of surface expression and of correlation with CD73 signature in 6 samples from FSGS, 8 from MCD, 10 from MGN and 8 from the amyloidosis group. In the CCR2 analysis, the control group consisted of 6 biopsies. Relevant clinical data were collected from electronic medical records (Table 1). Informed consent was waived because of the retrospective nature of the study, because of the fact that anonymous clinical data were used for analysis and because the study was performed on already existing formalin-fixed, paraffin-embedded tissue after completing the diagnostic process, as approved by the Ethic Committee of the University Medical Centre Mannheim, University of Heidelberg (Reference number: 2017-806R-MA).

### 4.2. Histology, Immunohistochemistry, Immunofluorescence and Electron Microscopy

The 1 µm serial sections were done on paraffin-embedded kidney biopsies and stained with hematoxylin/eosin (H/E), periodic acid-Schiff (PAS), Masson’s trichrome staining as well as standard routine immunohistochemistry for diagnostic purposes. Immunohistochemical and immunofluorescent staining of formalin-fixed paraffin embedded biopsies for were performed manually using rabbit anti-human CD73 monoclonal antibody (clone D7F9A, Cell Signaling, Frankfurt am Main, Germany) and mouse monoclonal anti-human CCR2 antibody (clone 7A7; Abcam, Cambridge, UK), rabbit polyclonal anti-human antibody to nephrin (Abcam, Cambridge, UK) and rabbit anti-human podocin antibody (Sigma, Saint Louis, MO, USA), according to manufacturer’s protocols. All of the biopsies were included in the analysis of podocyte CD73 expression by immunohistochemistry; for CCR2, paraffin sections with insufficient number of non-sclerotic glomeruli (<5) were excluded. To minimize the noise, antibody optimization for immunohistochemistry was performed using control (tonsil) tissue. The tissue sections were stained with Dako REAL Detection System Alkaline Phosphatase/RED Rabbit/Mouse kit (Agilent, Santa Clara, CA, USA) and Avidin/Biotin Blocking Kit (Vector laboratories, Burlingame, CA, USA). Antigen retrieval was performed for 20 min using Dako Target Retrieval solution (equivalent citrate buffer, pH 6.0). Primary antibodies were incubated for 60 min in DAKO antibody diluent with 2% dry milk at 37 °C and detected using the Dako REAL Link, biotinylated secondary antibodies (included in the kit), and Dako REAL Streptavidin Alkaline Phosphatase (AP). The reaction was visualized by a RED chromogen (included in the kit). The slides were counterstained with hematoxylin, dehydrated, mounted using Aquatex (Merck, Darmstadt, Germany) and cover-slipped. For immunofluorescence, CCR2/CD73 co-expression was evaluated in total of 22 podocytes from all podocyte lesion groups. CD73 was stained with Alexa Flour TM 488 Tyramide SuperBoost TM Kit, Streptavidin (Invitrogen, Thermo Fisher, Waltham, MA, USA) using secondary biotinylated anti-mouse antibody (Biotium, Fremont, CA, USA). Secondary antibodies for the immunofluorescence were donkey anti-mouse CF 594 and donkey anti-rabbit CF 594 (both from Biotium, Fremont, CA, USA).

For electron microscopy, biopsies were post-fixed with 1% osmium tetroxide (Roth, Karlsruhe, Germany), embedded in Agar 100 resin (Agar Scientific, Stansted, UK), cut at 90 nm thickness and stained with 1% uranyl acetate (Serva, Heidelberg, Germany). Micrographs were taken on transmission electron microscope JEM-1400 (Joel, Freising, Germany).

### 4.3. Light Microscopy and Morphometric Analyses

Image acquisition and analysis was done using the PreciPoint scanning microscope M8 with Olympus PlanCN 40×/0.65 objective and MicroPoint software (v.5 May 2016; PreciPoint, Freising, Germany). Semiquantitative evaluation of podocyte CD73 surface expression in immunohistochemical staining was assessed using a 0–3 scoring scale (0—no expression; 1—focal segmental positivity (<50% podocytes positive in <50% glomeruli); 2—focal global (>50% podocytes positive in <50% glomeruli); 3—diffuse (any percentage of podocytes positive in >50% glomeruli). CD73 positive and negative podocytes were analyzed for CCR2 co-expression using an intensity-based semiquantitative scoring system (0—no expression, 1—weak expression, 2—moderate expression and 3—strong expression, according to biopsy-internal CCR2 positive control—endothelium and infiltrating immune cells).

Evaluation was performed independently by two pathologists (SP and ZP) in a blinded manner.

### 4.4. Confocal Microscopy

Images of CD73 (Alexa Fluor 488), CCR2 (Alexa Fluor 594) and nuclei (DAPI) of podocytes were acquired sequentially with the Olympus FV1000 motorized confocal microscope equipped with an HeNe 594 nm, Argon 488 nm and a UV diode 405 nm laser and photomultiplier tubes (PMTs) controlled by the FluoView software, using the 60×/1.35 UPlanSApo objective. The 12-bit images with 512 × 512 pixel frames and pixel size of 0.41 µm for overviews and 0.10 µm for high resolution were acquired by keeping all the settings on the microscope constant. The images were further semi-automatically processed and analyzed using an in-house developed ImageJ (http://rsbweb.nih.gov/ij) macro (accessed date: 15 December 2020). Shortly, images were segmented for individual podocytes, threshold was applied, and mean intensity measured, statistically analyzed and plotted. All the images were processed with constant settings and displayed with the same pseudo-color equally linearly adjusted for individual channels on merged images.

### 4.5. Statistical Analyses

Statistical analyses were performed using GraphPad Prism v. 8.4.3 software (GraphPad Software, Inc., La Jolla, CA, USA). All data are reported as mean ± SEM. Statistical differences were calculated using paired or unpaired *t*-test as well as Pearson correlation analysis, as indicated.

## Figures and Tables

**Table 1:** Clinical parameters in control and podocyte damage groups.

	Control	MCD	FSGS	MGN	Amyloidosis
***n***	10	8	7	12	12
**Age (range)**	61.5 * (19–74)	46.5 (20–65)	44 (24–80)	55.5 (24–80)	60 (52–75)
**Gender (*n* = females, %)**	7 (70)	5 (62.5)	5 (71.4)	3 (25)	1 (8.3)
**Hypertension f (*n*, %)**	4 (40)	5 (62.5)	1 (14.3)	4 (33.3)	2 (16.67)
**Diabetes mellitus (*n*, %)**	3 (30)	0 (0)	1 (14.3)	2 (16.67)	0 (0)
**Creatinine, mg/dL (range)**	1.01 * (0.68–2.8)	0.85 (0.64–1.48)	0.88 (0.71–2.7)	0.76 (0.5–3.88)	1.88 (0.76–3.77)
**Proteinuria, g/24 h (range)**	0.36 * (0.1–0.86)	10.65 (3.2–24)	9.4 (5–43)	3.12 (0.2–15)	6.75 (2.63–16)

MCD—minimal change disease; FSGS—focal segmental glomerulosclerosis; MGN—membranous glomerulonephritis (primary and secondary). * Data are presented as median value (with range or percentage in brackets, as indicated).

## Data Availability

The data presented in this study are available on request from the corresponding author. The data are not publicly available due to ethical issues.

## Supplementary Materials

The following are available online at https://www.mdpi.com/article/10.3390/ijms22147642/s1.
