# Supplementary figures and images for: CD73 Overexpression in Podocytes: A Novel Marker of Podocyte Injury in Human Kidney Disease
Source: Int J Mol Sci. 2021 Jul 16;22(14):7642. doi: 10.3390/ijms22147642 (PMC8304086; doi:10.3390/ijms22147642)

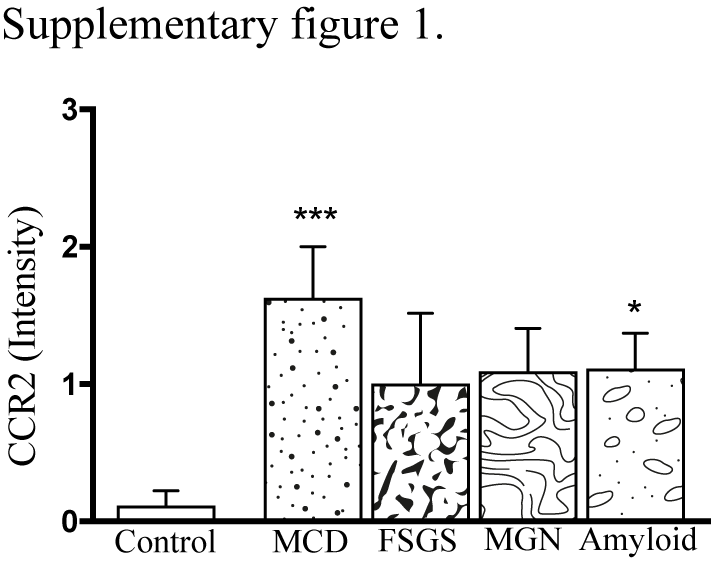

Supplement: Supplementary file 1 [file ijms-22-07642-s001.zip › ijms-1282911-supplementary.tif]
